# Radiomics and Artificial Intelligence for the Diagnosis and Monitoring of Alzheimer’s Disease: A Systematic Review of Studies in the Field

**DOI:** 10.3390/jcm12165432

**Published:** 2023-08-21

**Authors:** Roberta Bevilacqua, Federico Barbarossa, Lorenzo Fantechi, Daniela Fornarelli, Enrico Paci, Silvia Bolognini, Cinzia Giammarchi, Fabrizia Lattanzio, Lucia Paciaroni, Giovanni Renato Riccardi, Giuseppe Pelliccioni, Leonardo Biscetti, Elvira Maranesi

**Affiliations:** 1 Scientific Direction, IRCCS INRCA, 60124 Ancona, Italy; r.bevilacqua@inrca.it (R.B.); f.barbarossa@inrca.it (F.B.); s.bolognini@inrca.it (S.B.); c.giammarchi@inrca.it (C.G.); f.lattanzio@inrca.it (F.L.); e.maranesi@inrca.it (E.M.); 2 Unit of Nuclear Medicine, IRCCS INRCA, 60127 Ancona, Italy; l.fantechi@inrca.it (L.F.); d.fornarelli@inrca.it (D.F.); 3 Unit of Radiology, IRCCS INRCA, 60127 Ancona, Italy; e.paci@inrca.it; 4 Unit of Neurology, IRCCS INRCA, 60127 Ancona, Italy; l.paciaroni@inrca.it (L.P.); g.pelliccioni@inrca.it (G.P.); 5 Clinical Unit of Physical Rehabilitation, IRCCS INRCA, 60127 Ancona, Italy; g.riccardi@inrca.it

**Keywords:** radiomics, artificial intelligence, older people, Alzheimer, systematic review, diagnosis, MCI, machine learning, deep learning

## Abstract

The use of radiomics and artificial intelligence applied for the diagnosis and monitoring of Alzheimer’s disease has developed in recent years. However, this approach is not yet completely applicable in clinical practice. The aim of this paper is to provide a systematic analysis of the studies that have included the use of radiomics from different imaging techniques and artificial intelligence for the diagnosis and monitoring of Alzheimer’s disease in order to improve the clinical outcomes and quality of life of older patients. A systematic review of the literature was conducted in February 2023, analyzing manuscripts and articles of the last 5 years from the PubMed, Scopus and Embase databases. All studies concerning discrimination among Alzheimer’s disease, Mild Cognitive Impairment and healthy older people performing radiomics analysis through machine and deep learning were included. A total of 15 papers were included. The results showed a very good performance of this approach in the differentiating Alzheimer’s disease patients—both at the dementia and pre-dementia phases of the disease—from healthy older people. In summary, radiomics and AI can be valuable tools for diagnosing and monitoring the progression of Alzheimer’s disease, potentially leading to earlier and more accurate diagnosis and treatment. However, the results reported by this review should be read with great caution, keeping in mind that imaging alone is not enough to identify dementia due to Alzheimer’s.

## 1. Introduction

Nowadays, radiomics can be considered an emergent field of research, possibly allowing for the in-depth comprehension of diseases’ etiology and evolution. For radiomics, it is intended that the mineable data are extracted by converting clinical images to quantitative features using characterization algorithms [1], with the aim of identifying prognostic and predictive biomarkers of disorders. More in detail, radiomics allows for the extraction and analysis of a large number of quantitative features from medical images, such as magnetic resonance imaging (MRI) and positron emission tomography (PET) scans, echography and computerized tomography (CT), providing detailed information from medical images using mathematical and machine learning methods to explore possible ties with biology and clinical outcomes. In this way, a radiomics approach might help clinicians provide useful insights about the prognosis and response to treatment, thus representing a potential non-invasive method of precision medicine in the era of artificial intelligence (AI) [2].

From a general point of view, radiomics, given its intrinsic capacity of obtaining and organizing a huge number of data, seems to be particularly suited for studying multifactorial and complex diseases, and, not by chance, it has been mostly investigated in the oncology field [3,4,5,6,7]. Specifically, this new approach has been widely applied mainly in solid cancers [8,9,10], since data from histopathology and immunohistochemistry as well as from genomics were largely available, proving the expected heterogeneity of diseases at both cellular and molecular levels [11].

Recently, from the oncology field, given its power in the high-dimensional data mining of radiological features and its correlation with aging progression [12,13] and clinical endpoints [14], radiomics application is expected to spread in other multifactorial diseases where new signatures are difficult to be identified, including neurological ones. In this regard, recently, radiomics has shown interesting applications in the neurology field, highlighting promising results in differential diagnosis [15] among causes of cognitive impairment and in the prediction of the conversion from mild cognitive impairment (MCI) to Alzheimer dementia. In particular, Alzheimer’s disease (AD) is known to be the most common neurodegenerative disease. It has been estimated that over 46 million people live with dementia worldwide, increasing to 131.5 million by 2050 [16]. Therefore, there is an urgent need for biomarkers to be used for screening, diagnosis, prognosis and therapy response to control the societal impact of the disease.

In order to understand a vast plethora of data for the provision of the predictive models, AI algorithms represent elective strategies to be applied for the analysis of radiomic data, especially by adopting Deep Learning (DL) approaches based on neural networks (NN) that are able to deal with a huge number of computational parameters to be processed through high-performance technologies. Even if evidence from the literature shows that data-driven deep radiomic models have a better performance than humans in understanding diseases’ pathways, the method of the adoption of radiomic biomarkers still remains a gap to be solved to ensure their clinical uptake through efforts for standardization, the harmonization of different approaches and the reproducibility of the radiomic data collection in the different clinical settings [17]. 

The aim of this paper is to provide a systematic analysis of the studies that have included the use of radiomics from MRI and PET and AI for the diagnosis and monitoring of AD in order to improve the clinical outcomes and quality of life of older patients.

## 2. Materials and Methods

### 2.1. Literature Search and Study Selection

The methodology of this systematic review was based on the Preferred Reporting Items for Systematic Reviews and Meta-Analyses (PRISMA) guidelines [18], with the main aim of analyzing the impact of radiomic analysis and AI to diagnose and monitor AD and its progression.

We used the PICO framework (population, intervention, comparator and outcome) as follows:

P: Alzheimer patients

I: Diagnosis through artificial intelligence 

C: Mild cognitive impairment and healthy subjects

O: Differential diagnosis

A systematic review of the literature was conducted in February 2023. The data were collected from PubMed, Embase and Scopus, analyzing manuscripts and articles of the last 5 years (from January 2018 to January 2023) in order to obtain the latest evidence in the field. The inclusion criteria are as follows: (1) prospective or retrospective studies; (2) Alzheimer’s disease and cognitive impairment in people aged ≥65 years; (3) use of radiomics to discriminate between Alzheimer’s and mild cognitive impairment in the older population; (4) machine/deep learning classification in terms of accuracy, specificity, sensitivity, area under the curve of the received operating characteristic (AUC-ROC), positive predictive value (PPV), negative predictive value, recall and F1-score. Systematic and narrative reviews were excluded. Based on consultation with the multidisciplinary research team, multi-modal intervention studies were searched using the following search terms, and the combination thereof: radiomic*, elderly, Alzheimer, cognitive impairment, texture analysis, neurological status, frailty and sarcopenia. The full search strings are provided in Table 1. After the preliminary search, 1679 articles resulted from PubMed, 213 resulted from Embase and 372 resulted from Scopus. The findings were analyzed and screened by four experts of the team, a bioengineer, a clinical neurologist, a psychologist and a nuclear medicine physician. In particular, the four reviewers independently analyzed the titles and abstracts retrieved from the search in order to determine if they met the predefined inclusion criteria. The full text articles were subsequently analyzed. The first screening was based on the analysis of the title and the abstract of the findings. After the first step, 45 articles resulted from PubMed, 12 resulted from Embase and 9 resulted from Scopus. A second screening was based on a deduplication analysis of the findings. After this step, 18 papers were included from PubMed, 1 was included from Embase and 1 was included from Scopus. An additional researcher, with a background in biomedical engineering, confirmed the accuracy of the papers selection and screened for any possible omission.

### 2.2. Data Collection

**Figure 1:**
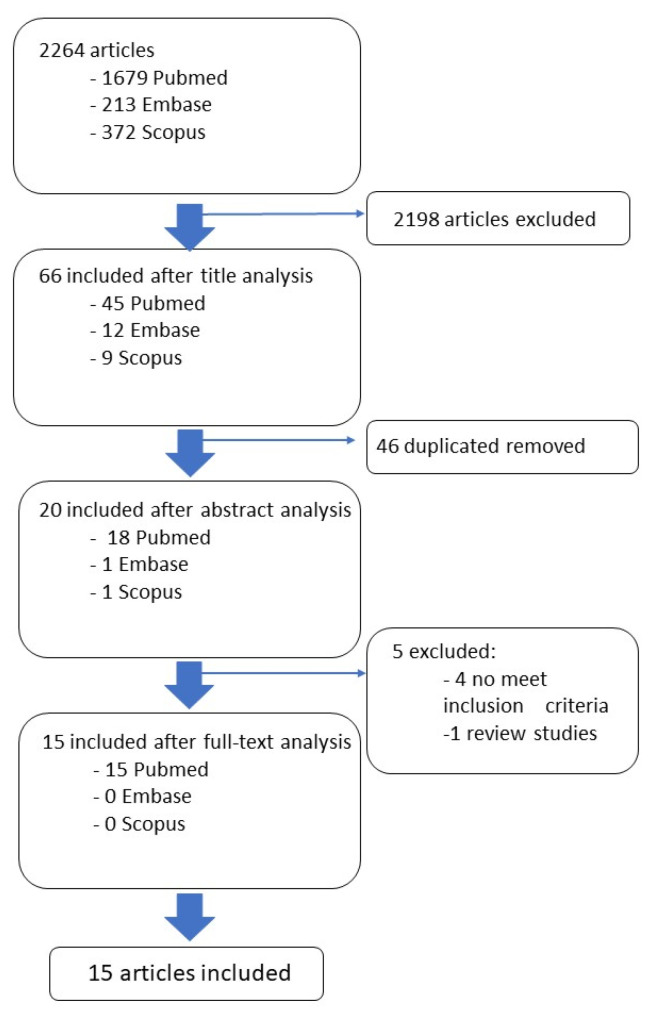
Descriptive analysis of the included clinical studies.

After the screening based on the inclusion/exclusion criteria conducted on the full text articles, the studies were selected as follows: 15 from PubMed, 0 from Embase and 0 from Scopus. Figure 1 shows the flowchart search strategy applied.

## 3. Results

A total of 15 papers were included [19,20,21,22,23,24,25,26,27,28,29,30,31,32,33]. The findings reported in this section are organized under macro-concept areas of interest.

### 3.1. Study Quality Evaluation

The quality evaluation of 15 population-based studies was performed based on the Newcastle Ottawa Scale (NOS) scale. The NOS is designed to assess the quality of non-randomized studies [34], such as case-control and cohort studies (all the studies included in this systematic review belong to this category). The Newcastle Ottawa Scale focuses on three main areas: the selection of the study groups, the comparability of the groups and the assessment of the outcome or exposure. The final score was settled when three authors reached an agreement after repeated review and analysis. Of the fifteen studies considered, the NOS score ranged from five to a maximum of nine (Table 2).

### 3.2. General Characteristics of the Study Population

In 8 out of 15 studies [20,21,22,23,28,29,31,33], the data used were retrieved from a public repository, 6 were from the Alzheimer’s Disease Neuroimaging Initiative (ADNI) database and 2 were from the Open Access Series of Imaging Studies (OASIS) [21,22]. In the remaining seven studies [19,24,25,26,27,30,32], original data were collected from human participants hospitalized in different healthcare structures. The 15 studies had an average sample size of 396.1, ranging from the smallest sample size of 86 [18] to the largest sample size of 1650 [20]. All the studies focus on older people, with a mean age of 71.3 (±8.87) years in the AD group, 75.8 (±8.9) years in the Mild Cognitive Impairment (MCI) group and 69.5 (±7.82) in the healthy control (HC). There were 1137 males and 1106 females in the AD group, 1188 males and 874 females in the MCI group and 1187 males and 1337 females in the HC group. The Mini-Mental State Examination and Clinical Dementia Rating are the most common neuropsychological assessment tests used to evaluate the cognitive and functional abilities of patients. Mini-Mental State Examination values are reported in 14 out of 15 studies [19,20,22,23,24,25,26,27,28,29,30,31,32,33], and a Clinical Dementia Rating is calculated in 7 out of 15 [21,22,23,24,27,28,30]. Other tests like the Montreal Cognitive Assessment, Auditory Verbal Learning Test, Alzheimer’s Disease Assessment Scale, Activities of Daily Living Scale and Geriatric Depression Scale are adopted. Table 3 summarizes the imaging method adopted in each study, the type of imaging evaluation, the AI algorithm adopted and the performance metrics used to check the classification/prediction performance of each ML/DL model, and Table 4 shows the characteristics of the included studies.

### 3.3. Radiomic Features

Radiomic features are extracted directly after segmentation from the area of interest. All studies used automatic feature extraction methods. The number of features calculated varies from a minimum of 35 [21] to 3360 [25]. Radiomic features can be broadly categorized into five classes: first-order features, shape-based features, texture features, wavelet-based features, and deep learning-based features. First-order features, or statistical features, were used in [19,20,21,23,24,27] to describe the basic statistics of the voxel intensity distribution within a region of interest, such as the mean, median, variance, skewness, and kurtosis. Shape-based features describe the geometric properties of the region of interest (ROI), such as the volume, surface area, and compactness. Shape features are calculated in all papers. Texture features capture the spatial distribution of voxel intensities within the ROI and provide information on the heterogeneity, coarseness, and complexity of the tissue. Texture features are extracted using different methods, such as the gray-level co-occurrence matrix (GLCM), gray-level run-length matrix (GLRLM), gray-level size zone matrix (GLSZM), gray-level dependence matrix (GLDM) and neighboring gray-tone difference matrix (NGTDM), as accomplished in all reported studies. Wavelet-based features use wavelet transforms to decompose the image into multiple frequency bands and extract features from each band. Wavelet features are calculated in all the studies except for [19,20,31].

Finally, deep learning-based features are extracted by training a neural network on a large dataset of images to identify patterns and features that are relevant for the task at hand. These features can then be used to extract radiomic features from new images, as accomplished in [22,33]. 

Radiomics features are then reduced in dimensionality with different techniques: Least Absolute Shrinkage and Selection Operator (LASSO) was used in six studies [19,23,24,25,26,27], *t*-test statistical correlation analysis was adopted in three studies [23,28,29], Cox regression was used in two studies [26,28] and Fisher’s score was adopted in one [30].

### 3.4. Machine and Deep Learning Methods Applied to the Diagnosis of AD

Machine learning models can be pooled into two categories: support vector machine (SVM) and logistic regression (LR). In seven studies, an SVM classification model was built. In particular, the radial basis function was chosen as the SVM algorithm kernel [20,23,29,32,33], while a multi-function kernel (linear, polynomial and sigmoid) was involved in two studies [28,30] in order to evaluate the performance of each SVM model. On the other side, in three studies, an LR algorithm was used to build a diagnostic model [19,25,26]. Convolutional neural networks (CNN) were used as deep learning algorithms to perform AD diagnosis in three studies [21,22].

### 3.5. Performance Metrics

Different metrics were used to assess the performance of ML and DL models. All studies report metrics referring both to the test set and validation set. Accuracy is adopted in 12 out of 15 studies [20,23,24,25,26,27,28,29,30,31,32,33]. It was used individually only in one study [28], while in all the other studies, it was calculated in combination with other metrics. The most common metric is the area under the curve (AUC), used in 13 out of 15 studies [19,20,21,22,23,24,25,26,27,29,31,32,33], while sensitivity and specificity were used in 9 out of 15 studies [19,23,24,25,26,27,30,31,32,33]. The least used metrics for evaluating the model classification performance are precision (3 out of 15) [19,26,27], positive predictive value (PPV) and negative predictive value (NPV) (2 out of 15) [19,26], recall and F1 score (2 out of 15) [19,27].

### 3.6. Differential Diagnosis

In 9 out of 15 studies [20,21,22,25,26,27,29,31,32], radiomics analysis is used to perform a differential diagnosis between AD and HC. In detail, among these 10 articles, 4 of them [21,22,26,27] focus exclusively on the discrimination between AD and HC, while in the other research [20,25,29,31,32], the authors focused in parallel on radiomic analysis aimed at the discrimination between MCI and AD, AD and HC.

In [19,30,33], the main core is the differential diagnosis between Subjective cognitive decline (SCD) and HC. It is noteworthy that SCD is often considered a potential early indicator of cognitive impairment or dementia. However, SCD is not a diagnostic criterion for dementia or other cognitive disorders, as many individuals with SCD do not progress to develop dementia or cognitive impairment. 

In addition, in [28,29], relevance is given to progression from MCI to AD, while in [23], early-onset AD and late-onset AD against HC are classified. Indeed, a recent study has suggested considerable differences between early-onset and late-onset AD from a clinical point of view [35]; thus, we found it interesting to add this article within this review. For instance, early-onset AD patients show a faster and more severe disease progression compared to late-onset AD patients. They also present an unusual pattern of preserved memory function alongside cortical symptoms that affect language, visuospatial skills, and executive function, as reported by Cacace et al. [36]. Additionally, early-onset AD patients have less damage in the hippocampus but more severe damage in the neocortex. Furthermore, they display functional disruption between the hippocampus and middle frontal cortex, which distinguishes them from late-onset AD patients. On the other hand, in [24], the authors made a radiomic analysis to predict amyloid β peptide positivity and negativity. The amyloid β peptide status is crucial not only for diagnostic purposes but also for predicting the clinical course of patients in the early stage of AD. In particular, the Aβ status is associated with clinical deterioration and the transition to dementia in patients with mild cognitive impairment (MCI).

### 3.7. Brain Regions and Classification Results

In 14 out of 15 studies [19,20,21,22,23,24,25,26,27,28,29,30,32,33], regions of interest were extracted from MR images, while only in [31], Aβ PET was used to detect the accumulation of the beta-amyloid protein in the brain. For the identification of AD from HCs with the radiomics features of Aβ PET images, the authors obtained an AUC = 0.93 with the standard machine learning SVM method, while in all other studies using MRI, the AUC values ranged from 0.72 to 0.93. The segmentation of the MRI/PET image of the brain is aimed at extracting one or more anatomical areas of interest from which radiomic features are then calculated.

The hippocampus is the most interesting research area, with 6 out of 15 studies focusing on it [19,20,23,28,29,32]. In all of these studies, the hippocampus was taken singularly. For studies that take the hippocampus as the anatomical reference, AUC values ranging from 0.88 to 0.94 are obtained for studies that focus on the differentiation between AD patients and healthy subjects. Among these, the ADNI database was used in four papers [20,23,28,29], while in the remaining two studies [19,32], the sample was collected inside the healthcare structure. Individually, the amygdala [25] and corpus callosum [26] were also analyzed. For the study that refers to radiomic analysis of the amygdala, a satisfactory diagnostic performance in terms of accuracy (90%) in discriminating between AD and healthy subjects was reported, while slightly lower values were obtained with respect to the differentiation between patients with AD dementia and MCI. In this case, a database of patients internal to the referring hospital was used. As for the corpus callosum, the study investigating it provides a classification accuracy level between AD and healthy people of 79.2%. In this study, the referral patients were selected from within the healthcare center too. In two studies, gray matter, white matter and cerebrospinal fluid were segmented together [30,33]. For these two studies, the accuracy in differentiating between SCD or pre-clinical AD subjects on one hand and healthy subjects on the other hand reached over 89% in both [30,33].

In this case, in [33], the sample is retrieved from the ADNI repository, while the other study examines an in-house repository at the health institution. In the remaining four studies [21,22,27,31], researchers do not involve the segmentation of a specific anatomical region; rather, they perform training of their classifiers by whole-brain imaging. In [21], the authors achieved 93.9% accuracy in the differential analysis between AD and healthy subjects from the OASIS repository. In [22], an accuracy of 92.6% was achieved, in [27], one of 96.2% was achieved and in [31], the AUC was 0.93 for distinguishing AD from healthy subjects and 0.83 for the prediction of MCI conversion to AD. In this case, only in [27] was a local repository used, while the others adopted the OASIS database for the sample acquisition.

## 4. Discussion

In the current AD diagnostic criteria, cerebrospinal fluid (CSF), namely, CSF amyloid β 42 (Aβ 42) or Aβ 42/40, phosphorylated tau and total tau levels and/or PET biomarkers, i.e., amyloid PET and tau PET, were included [37,38]. However, these biomarkers are affected by some limitations. In particular, CSF biomarkers can be assessed only after having performed a lumbar puncture, which is considered a quite invasive procedure, and amyloid- PET and tau PET are expensive and not always available. Therefore, growing efforts are being made by researchers in order to identify reliable, easily available and non-invasive AD biomarkers. In this regard, radiomics might be an interesting option.

The present systematic review included all studies focused on the use of radiomics in the AD field, of which 14 [19,20,21,22,23,24,25,26,27,28,29,30,32,33] considered data from MRI, and only 1 [31] was based on data analysis from amyloid PET imaging. Globally considered, the results showed a very good performance of this approach in the differentiating AD patients—both at the dementia and pre-dementia phases of the disease—from HC patients. Indeed, the majority of studies reported a very high accuracy, with an AUC ranging from 80 to 95%, approximately. It is noteworthy that, according to current AD diagnostic criteria, MRI is considered a progression biomarker, rather than a diagnostic one, because of the suboptimal diagnostic performance, mainly in the view of early AD diagnosis, in terms of sensitivity and specificity, compared to the CSF and tau/amyloid PET biomarkers. Interestingly, some radiomics studies, based on MRI and focused on early AD diagnosis, reported such impressive results in terms of the accuracy of the prediction of MCI conversion to AD. In [24], T1 and T2 FLAIR radiomics data, together with demographic features, were able to distinguish between amyloid-positive and amyloid-negative patients, as classified by means of amyloid PET, with a quite satisfactory accuracy (AUC for test = 0.79; AUC for validation = 0.76). Despite this diagnostic, the performance is still clearly inferior compared to that of the CSF and PET biomarkers. These preliminary results could be considered encouraging, but they need to be validated by further investigations. 

Interesting findings also come from studies exploring the ability of radiomics in differentiating patients with subjective cognitive decline (SCD) and/or pre-clinical AD from controls [30,33]. Subjective Cognitive Decline may represent a very early phase of AD, which precedes MCI; however, not all SCD subjects will develop AD during follow-up. In one of those two studies, SCD subjects were included, but they were not well characterized due to the lack of clinical follow-up data and/or assessment by means of AD-validated biomarkers at baseline [30]. Considering these limitations, no conclusive statement about the findings from this study with respect to early AD diagnosis will be made. The second investigation in this field, by Jiang and colleagues [33], included a large cohort of well-characterized, cognitively unimpaired subjects, who were divided in two groups (amyloid-positive and amyloid-negative), according to the amyloid PET status. In this investigation, a Deep Learning Radiomics method was used, obtaining a very high diagnostic accuracy (89.85 ± 1.12%) in differentiating amyloid-positive cognitively normal subjects (i.e., pre-clinical AD patients according to the current diagnostic criteria) from amyloid-negative healthy subjects (i.e., persons without any clinical and/or biological evidence of a neurodegenerative disorder). If confirmed by future investigations, those results might open a new era for the non-invasive assessment of aged healthy people in the view of early AD diagnosis, making it crucial to enroll subjects in similar clinical trials in order to test new disease-modifying therapies. 

Also, the study on radiomics applied to amyloid-PET imaging [31] showed a very satisfactory accuracy (AUC = 0.93) in differentiating patients with AD from patients from normal controls and in predicting MCI conversion to AD dementia. However, in our opinion, the application of radiomics to amyloid-PET cannot be easily implemented in clinical practice in the future, due to the different availability of PET machines in hospitals and the absence of a standardization of a radiomics approach in this field. Despite the promising findings reported in papers included in this review, radiomics studies in the AD field are still affected by a great heterogeneity in terms of patients’ enrolment criteria, region of interest and other technical issues. Those aspects have impeded us from performing a rigorous meta-analysis; thus, we conducted a qualitative systemic review of the studies on this topic. Since radiomics is nowadays a quite complicated and time-consuming approach to be applicable in clinical practice for AD, larger prospective studies are needed to clarify its potential usefulness in the field.

## 5. Conclusions

Radiomics and artificial intelligence have the potential to help in the early detection of and diagnosis of Alzheimer’s by analyzing medical images and other data to identify patterns that contribute to developing the disease. In summary, radiomics and AI can be valuable tools for diagnosing and monitoring the progression of Alzheimer’s disease, potentially leading to earlier and more accurate diagnosis and treatment. However, the most critical limitation of this review is that all included articles do not have pathological confirmation of the clinical diagnosis. Although all patients included in the databases were meticulously screened by clinical experts in defining the pathology (MCI or Alzheimer’s disease), no further biological references were taken in the reviewed articles, and only imaging information was considered. In this regard, the results reported by this review should be read with great caution, keeping in mind that imaging alone is not enough to identify dementia due to Alzheimer’s disease. It is necessary to conduct a multi-omics analysis that considers a combination of biomarkers to increase the degree of accuracy with which an early diagnosis of the pathology can be made. It is for this reason that, for clinical practice, it is still difficult to rely solely on the use of imaging to diagnose pathology. However, the review presents interesting results for research purposes that pave the way for future developments in artificial intelligence related to Alzheimer’s identification.

## Figures and Tables

**Table 1:** Adopted search strategy.

Order of Search	Terms
1	Elderly AND Radiomics AND Alzheimer
2	Radiomics AND Elderly AND Cognitive impairment
3	Texture analysis AND Elderly AND Cognitive impairment
4	Radiomics AND Elderly AND Neurological status
5	Radiomics AND Elderly AND Frailty
6	Radiomics AND Sarcopenia

**Table 2:** Scores of quality assessment of the included studies according to the Newcastle Ottawa Scale. * indicates that the item requirement is satisfied; 0 indicates that the item requirement is not satisfied.

	Selection				Comparability		Outcome			
Study	Adequate Case Definition	Representativeness of the Cases	Selection of Controls	Definition of Controls	Main Factor	Additional Factor	Ascertainment of Exposure	Same Ascertainment for Cases and Controls	Non-Response Rate	Total (9/9)
Feng et al. [19]	*	*	*	*	*	0	*	*	*	8
Zheng et al. [20]	*	*	*	*	*	0	*	*	0	7
Chaddad et al. [21]	*	*	0	*	*	0	*	*	0	6
Chaddad et al. [22]	*	*	0	*	*	0	*	*	0	6
Du et al. [23]	*	*	*	*	*	*	*	*	*	9
Kim et al. [24]	*	*	*	*	*	0	*	*	*	8
Feng et al. [25]	*	*	*	*	*	0	*	*	*	8
Feng et al. [26]	*	*	*	*	*	*	*	*	0	8
Liu et al. [27]	*	*	*	*	*	*	*	*	*	9
Li et al. [28]	*	*	*	0	*	0	*	*	0	5
Zhao et al. [29]	*	*	*	0	*	0	0	*	*	6
Wu et al. [30]	*	*	0	0	*	0	*	*	0	6
Ding et al. [31]	*	*	*	*	*	0	*	*	*	8
Feng et al. [32]	*	*	*	*	*	0	*	*	*	8
Jiang et al. [33]	*	*	*	0	*	0	*	*	*	7

**Table 3:** Studies grouped by macro-categories. For each article, the following is reported: the state or condition of the disease, the method and evaluation of each imaging technique, the AI algorithm adopted for the classification and prediction and the metrics for evaluating the model’s performance.

Study	State/Condition of Disease	Imaging Method	Imaging Evaluation	AI Algorithm	Performance Measures
Feng et al. [19]	Amnestic mild cognitive impairment	Magnetic resonance	Segmentation and feature extraction with LASSO	Logistic regression	AUC, sensitivity, specificity, precision, recall, F1-score
Zheng et al. [20]	Mild cognitive impairment	Magnetic resonance	Segmentation and HRF feature extraction	Support vector machine	AUC, accuracy
Chaddad et al. [21]	Alzheimer disease	Magnetic resonance	Entropy feature extraction	Convolutional neural network, random forest	AUC
Chaddad et al. [22]	Alzheimer disease	Magnetic resonance	CNN-derived feature extraction	Convolutional neural network, random forest	AUC
Du et al. [23]	Early and late onset of Alzheimer disease	Magnetic resonance	Segmentation and feature extraction	Support vector machine	AUC, accuracy
Kim et al. [24]	Amyloyd-positive and -negative	Magnetic resonance	Segmentation and feature extraction	-	AUC, sensitivity, specificity
Feng et al. [25]	Mild cognitive impairment	Magnetic resonance	Segmentation and feature extraction	Logistic regression	AUC, Accuracy
Feng et al. [26]	Alzheimer disease	Magnetic resonance	Segmentation and feature extraction	Logistic regression	AUC, accuracy, sensitivity, specificity, precision,
Liu et al. [27]	Alzheimer disease	Magnetic resonance	Segmentation and feature extraction	Logistic regression, K Nearest Neighbor, Support vector machine	Accuracy
Li et al. [28]	Mild cognitive impairment, Alzheimer disease	Magnetic resonance	Segmentation and feature extraction	Support vector machine	Accuracy
Zhao et al. [29]	Mild cognitive impairment	Magnetic resonance	Segmentation and feature extraction	Support vector machine, linear discriminant analysis	AUC, accuracy, sensitivity, specificity
Wu et al. [30]	Subjective cognitive decline	Magnetic resonance	Segmentation and feature extraction	Support vector machine, random forest	AUC, accuracy, sensitivity, specificity
Ding et al. [31]	Mild cognitive impairment, Alzheimer disease	Positron emission tomography	Feature extraction	Support vector machine	AUC
Feng et al. [32]	Mild cognitive impairment	Magnetic resonance	Segmentation and feature extraction	Support vector machine	AUC, accuracy, sensitivity, specificity
Jiang et al. [33]	Alzheimer disease	Magnetic resonance	Derived feature extraction	Convolutional neural network, support vector machine	AUC, accuracy, sensitivity, specificity

**Table 4:** Descriptive analysis.

	Population		Methods	Aim of the Study	Results
	Participants in AD Groups	Participants in the HC Group			
Feng Qi [19]	n = 42 older adults, 18 M/24 F, Age 64.17 ± 10.57 years	n = 44 older adults, 20 M/24 F, Age 65.43 ± 9.70 years	After the segmentation of the left and right hippocampus, features are extracted and selected using LASSO to build two classification models based on logistic regression. N. of radiomics features: 45 Brain area: Hippocampus Raw image: MRI ML algorithm: LR	Primary: to identify the radiomic biomarkers of the hippocampus for building the classification models in SCD/AD diagnosis.	The study reports ML classification results divided between the left and right hippocampus: AUC (L/R): 0.79/0.76 Sensitivity: (L/R) 0.54/0.69 Specificity: (L/R) 0.71/0.71 Precision: (L/R) 0.64/0.69 Recall(L/R): 0.54/0.69 F1-score (L/R): 0.58/0.69
Zheng et al. [20]	n = 283 older adults, 152 M/131 F, Age 74.91 ± 7.70 years	MCI group n = 764 older adults, 447 M/317 F, Age 72.96 ± 7.70 years HC group n = 603 older adults, 277 M/326 F, Age 73.46 ± 6.17	After calculating the HRFs of the intensity, shape and textural features from each side of the hippocampus in MRI, the consistency of the HRFs calculated from seven different hippocampal segmentation methods was validated, as well as the performance of the machine learning–based classification of AD vs. HC. N. of radiomics features: 55 Brain area: Hippocampus Raw image: MRI ML algorithm: SVM	Primary: identification of the optimal segmentation methods. Secondary: discrimination of AD among HC.	Regarding the classification of AD and MCI, the bilateral HRFs exhibited acceptable results for all of the involved segmentation. AUC: 0.88 Accuracy: 83.9%
Chaddad et al. [21]	n = 100 older adults, Age > 60	n = 98 older adults, Age > 60	Once the images are pre-processed, a convolution and a max pooling allow for the extraction of conditional entropy features. Such features are used to train and test a 100-trees random forest algorithm and a CNN to distinguish between AD and HC. N. of radiomics features: - Brain area: - Raw image: MRI ML/DL algorithm: CNN/RF	Primary: The goal of this experiment is to classify between AD and HG subjects from MRI data.	The highest overall accuracy, 93.6%, is obtained from an RF classifier combining the three conditional entropy features.
Chaddad et al. [22]	n = 100 older adults, 41 M/59 F, Age 77 years	n = 135 older adults, 38 M/97 F, Age 71 years	MRI volumes are first processed to label subcortical areas from which radiomics features are extracted. A significance test is performed to identify features that show significant differences between AD and HC. Novel CNN-derived radiomic features based on the entropy are proposed to improve classification. These features are then used as the input to an RF model to classify AD and HG subjects. N. of radiomics features: 45 Brain area: Subcortical regions Raw image: MRI DL/ML algorithm: CNN/RF	Primary: Propose a novel technique, based on the entropy of convolutional feature maps, to characterize the local texture in a data-driven manner and to classify AD from HG through CNN.	Among subcortical regions, the hippocampus (AUC 81.19–84.09%) and amygdala (AUC 79.70–80.27%) regions have the greatest discriminative power. Entropy features derived from CNN show an AUC of 0.925 in discriminating AD from HG after testing RF.
Du et al. [23]	n = 72 older adults, 35 M/37 F, Age 66.12 ± 2.8 years	n = 78 older adults, 36 M/42 F, Age 70.6 ± 7.5 years	Hippocampal segmentation and feature extraction were performed; the LASSO method was used to select radiomic features. SVM models were constructed based on the identified features to distinguish early onset AD from young control subjects, late onset AD from old control subjects and early onset AD from late onset AD subjects. N. of radiomics features: - Brain area: L/R Hippocampus Raw image: MRI ML algorithm: SVM	Primary: build and validate radiomics models of the hippocampus for early onset AD and young controls, late onset AD and old controls and early onset AD and late onset AD.	SVM models have good performances. Early onset AD and HG AUC: 0.90 Accuracy: 77% Late onset AD and HG AUC: 0.94 Accuracy: 86% Early onset and late onset AUC: 0.87 Accuracy: 79%
Kim et al. [24]	Amyloid-positive older adults n = 166, 70 M/96 F, Age 71.9 ± 8.1 years	Amyloid-negative older adultsn = 182, 78 M 104 F, Age 70.9 ± 8.6 years	After multimodal MRI images were pre-processed, the brain region of interest was segmented, and radiomic features were extracted from each. Once radiomic features are selected through LASSO, the remaining features were used alone or in combination with baseline non-imaging predictors such as age, sex and ApoE genotype to predict amyloid positivity. N. of radiomics features: - Brain area: whole brain Raw image: MRI ML algorithm: -	Primary: Predicting amyloid positivity in patients with MCI. Secondary: compared the predictive value of a radiomics model with those of cortical thickness and non-imaging predictors	Amyloid positivity was predicted a using non-imaging model (a model based on features not derived from MRI) that had an AUC = 0.71. Among single MR-sequence models, T1 MRI showed an AUC of 0.71, and T2 MRI showed an AUC of 0.74. When T1 and T2 radiomics features were combined, the AUC for the test was 0.75
Feng Q. et al. [25]	n = 41 older adults, 16 M/25 F, Age 76.1 ± 8.7 years	MCI group n = 60 older adults, 43 M/17 F, Age 76 ± 8.4 years HC group n = 72 older adults, 26 M/46 F, Age 75.2 ± 4.7	Once images are pre-processed and segmented, radiomics features are extracted, and LASSO is used to reduce and select the features. Multivariate LR analysis was performed to build classification models followed by an internal validation. N. of radiomics features: 3360 Brain area: Amygdala Raw image: MRI ML algorithm: LR	Primary: build and validate comprehensive classification models based on amygdala radiomic features to discriminate AD, MCI and NC	LR analysis based on amygdala radiomic features achieves good performance for clinical applications among AD, SCD and HC groups. Accuracy AD vs. HG: 90% Accuracy AD vs. SCD: 81% Accuracy SCD vs. HG: 75%
Feng Q. et al. [26]	n = 78 older adults, 25 M/53 F, Age 69.18 ± 12.23 years	n = 44 older adults, 20 M/24 F, Age 65.43 ± 9.70 years	After manual segmentation, features are extracted from the region of interest and selected using LASSO. The logistic regression method is then applied to establish a classificationmodel for AD diagnosis. N. of radiomics features: 385 Brain area: Corpus callosum Raw image: MRI ML algorithm: LR	Primary: identify the CC radiomic features related to the diagnosis of AD and build and evaluate a classification model from NC.	Eleven features were selected from the using the LASSO model. Discrimination of AD from NC: AUC: 0.720 Sensitivity: 79.2% Specificity: 50.0% Accuracy: 68.4% Precision: 73.1% PPV: 73.1% NPV: 58.3%
Liu et al. [27]	n = 80 older adults, 42 M/38 F, Age 65	n = 80 older adults, 40 M/40 F, Age 64	For each patient, MRI images were segmented into 106 subregions, and radiomic features were extracted from each. Then, an analysis is conducted of radiomic features of specific brain subregions that were most related to AD. Based on the selective radiomic features from specific brain subregions, they built an integrated model using the best machine learning algorithm. N. of radiomics features: - Brain area: whole brain segmented regions Raw image: MRI ML algorithm: multiple	Primary: exploring optimal brain regions for AD radiomics diagnosis. Secondary: Evaluate the optimal ML algorithm to discriminate AD from HG.	The subregions most relevant to AD included the hippocampus, the inferior parietal lobe, the precuneus and the lateral occipital gyrus. Accuracy LR: 96.2% Accuracy KNN: 95.0% Accuracy SVM: 95.0%
Li et al. [28]	n = 165 older adults, 103 M/62 F, Age 75.3 ± 6.3 years	n = 32 older adults, 13 M/19 F, Age 76.2 ± 6.8 years	After segmentation, normalization and smoothing, radiomics features are extracted from MR images and selected through statistical analysis (Cox regression and *t*-test). The classification phase was accomplished using SVM models with three different linear, polynomial and sigmoid kernels N. of radiomics features: 215 Brain area: L/R Hippocampus Raw image: MRI ML algorithm: SVM	Primary: prove that radiomics features could be used to identify the fast and slow conversion from MCI to AD based on MRI images.	Classification accuracy using linear, polynomial and sigmoid kernels could achieve good discrimination in distinguishing MCI-to-AD fast and slow conversion. In particular, in terms of accuracy, SVM with a polynomial kernel shows the best results. Accuracy linear: 80.0%, Accuracy polynomial: 93.3% Accuracy sigmoid: 86.6%
Zhao et al. [29]	n = 583 older adults, 268 M/315 F, Age 69.74 ± 8.14 years	MCI group n = 773 older adults, 429 M/344 F, Age 69.85 ± 8.31 years HC group n = 587 older adults, 268 M/319 F, Age 67.87 ± 6.82	After the automatic segmentation of the hippocampal region, intensity-based features, shape-based features and texture-based features across eight wavelet-based frequency domains are extracted. The SVM model is built to classify the AD patients and NC. N. of radiomics features: 55 Brain area: Hippocampus Raw image: MRI ML algorithm: SVM, LDA	Primary: verify if hippocampal region features can serve as robust MRI markers for AD. Secondary: Diagnose AD through ML.	Multivariate classifier-based SVM analysis provided individual-level predictions for distinguishing AD patients HC with an accuracy of 88.21% and inter-site cross validation.
Wu et al. [30]	SCD group n = 103 older adults, 71 M/32 F, Age 68.4 ± 6.60 years	n = 132 older adults, 85 M/47 F, Age 67.23 ± 6.41 years	After brain sMRI segmentation and feature extraction, the *t*-test, autocorrelation, and Fisher score were performed for selecting the most relevant features. SVM was implemented to build a classification model, and a random forest (RF) was used as a comparison model. N. of radiomics features: 215 Brain area: Grey matter, white matter, cerebrospinal fluid Raw image: MRI ML algorithm: SVM, RF	Primary: propose a radiomic approach to detect neuropathological features in subjective cognitive decline (a high-risk preclinical stage in the progress of AD) subjects based on MRI images.	SVM showed good results in the classification of SCD from HC. Accuracy: 84.7% Sensitivity: 86.9% Specificity: 82.6%
Ding et al. [31]	n = 291 older adults, 173 M/118 F, Age 74.69 ± 7.26 years	MCI group n = 453 older adults, 272 M/181 F, Age 73.51 ± 6.64 years HC group n = 334 older adults, 167 M/167 F, Age 73.78 ± 6.01 years	After extraction of radiomics features and group difference statistical analysis, a nonlinear SVM model with a radial basis function kernel was adopted to predict over AD cases. Feature selection was introduced by combining the *t*-test and SVM-RFE. N. of radiomics features: 47 Brain area: 246 regions Raw image: PET ML algorithm: SVM	Primary: explore whether the radiomic features of PET images are used as predictors and provide a neurobiological foundation for AD. Secondary: Diagnose AD through ML	The results showed a high accuracy in distinguishing AD from HC and predicting the MCI conversion to AD. The classification outputs were found to be significantly associated with clinical measures like the apolipoprotein E genotype. AUC AD from HC: 0.93 AUC MCI to AD: 0.83
Feng et al. [32]	n = 38 older adults, 16 M/22 F, Age 71.7 ± 8.3 years	MCI group n = 33 older adults, 14 M/19 F, Age 70.6 ± 8.2 years HC group n = 45 older adults, 22 M/23 F, Age 68.2 ± 6.9 years	Region of interest is extracted after the segmentation of MR scans. Features are extracted and selected to combine the spatial and frequency characteristics. ANOVA was employed to evaluate the differences between the AD, SCD and HCgroups, and Spearman’s correlation coefficient was calculated to evaluate the relationships between the features and MMSE. The SVM model is built to perform a classification task. N. of radiomics features: 423 Brain area: L/R Hippocampus Raw image: MRI ML algorithm: SVM	Primary: test whether radiomic features in the hippocampus can be employed for the early classification of AD and SCD. Secondary: Distinguish AD from HC through ML.	The SVM model demonstrated that radiomic features allowed for distinguishing AD from HC with a satisfying performance. AUC: 0.93 Accuracy: 86.75% Specificity: 88.89% Sensitivity: 84.21%
Jiang et al. [33]	n = 181 older adults, 73 M/108 F, Age 76.3 ± 5.4 years	n = 228 older adults, 112 M/116 F, Age 71.8 ± 5.7 years	Once the MRI image is pre-processed, a basic DLR model pre-training is carried out through CNN. To obtain DLR features, global max pooling is used to extract the maximum value of each feature map (last convolutional layer of the DL model). Based on the above extracted features, SVM is used to distinguish AD from HC. N. of radiomics features: - Brain area: GM, WM, CSF Raw image: MRI ML/DL algorithm: CNN for DLR feature extraction/SVM for classification	Primary: propose a novel deep learning radiomics method for distinguishing cognitively normal adults at risk of AD from NC.	The DLR method achieved the best classification performance between AD and HC compared to other models (hippocampal/traditional radiomics/clinical): AUC: 0.904 Accuracy: 92.8% Sensitivity: 88.8% Specificity: 94.4%

AD = Alzheimer disease, AUC = Area under the curve, CC = Corpus callosum, CNN = Convolutional neural network, DLR = Deep learning radiomics, HC = Healthy control, HRF = Hippocampal radiomic features, LASSO = Least Absolute Shrinkage and Selection Operator, LDA = Linear discriminant analysis, LR = Logistic regression, MCI = Mild cognitive impairment, MR = Magnetic resonance, PPV = Positive predictive value, NPV = Negative predictive value, RF = Random forest, RFE = Recursive feature elimination, SVM = Support vector machine.

## Data Availability

The datasets generated, used and analyzed during the trial and its preceding pilot trial are or will be available from the corresponding author upon reasonable request.
